# Lymphocyte and neutrophil count combined with intestinal bacteria abundance predict the severity of COVID-19

**DOI:** 10.1128/spectrum.03027-23

**Published:** 2023-12-13

**Authors:** Liuying Chen, Zhongwei Yin, Dan Zhou, Xin Li, Cheng Yu, Chang Luo, Yu Jin, Lei Zhang, Jun Song, Leo Rasche, Hermann Einsele, Lei Tu, Xiang Zhou, Tao Bai, Xiaohua Hou

**Affiliations:** 1 Division of Gastroenterology, Union Hospital, Tongji Medical College, Huazhong University of Science and Technology, Wuhan, China; 2 Division of Cardiology, Hubei Key Laboratory of Genetics and Molecular Mechanisms of Cardiological Disorders, Tongji Hospital, Tongji Medical College, Huazhong University of Science and Technology, Wuhan, China; 3 Department of Paediatrics, Union Hospital, Tongji Medical College, Huazhong University of Science and Technology, Wuhan, China; 4 Ultrasonic Department, Union Hospital, Tongji Medical College, Huazhong University of Science and Technology, Wuhan, China; 5 Department of Internal Medicine II, University Hospital Würzburg, Julius-Maximilian University of Würzburg, Würzburg, Germany; Brigham and Women's Hospital, Boston, Massachusetts, USA

**Keywords:** COVID-19, lymphocytes, neutrophils, bacteria

## Abstract

Early identification of severe 2019 coronavirus disease (COVID-19) cases is of great significance. We included 92 COVID-19 patients and 22 healthy controls and collected their laboratory test data and stool for metagenomic sequencing analysis. We found that the proportion of severe COVID-19 cases was significantly increased in patients with reduced lymphocytes and elevated neutrophils and had unique intestinal microbiota. *Pseudothermotoga hypogea* and *Lachnospiraceae bacterium GAM79* were the species of bacteria that markedly decreased in COVID-19 patients compared to control individuals. For COVID-19 patients, *Enterococcus* sp. *DA9*, *Caldicellulosiruptor obsidiansis*, and *Enterococcus faecium* were the species of bacteria that significantly altered among patients with normal lymphocytes and neutrophils and patients with decreased lymphocytes and/or increased neutrophils, respectively. Fecal abundances of *Enterococcus* sp. *DA9*, *Prevotella intermedia*, and *Bifidobacterium dentium* were correlated with COVID-19 disease severity. Decreased lymphocytes and increased neutrophils, combined with decreased *Bifidobacterium dentium* and *Prevotella intermedia*, and increased *Enterococcus* sp. *DA9* have the highest power to predict severe cases of COVID-19 patients (area under receiver operating characteristic curve: 0.79). In conclusion, changes in the abundance of intestinal bacteria can reflect the severity of COVID-19 disease, and the combination with lymphocytes and neutrophils might be prognostic factors of COVID-19.

**IMPORTANCE:**

The 2019 coronavirus disease (COVID-19) patients had a unique profile of gut bacteria. In this study, we characterized the intestinal bacteria in our COVID-19 cohorts and found that there was an increased incidence of severe cases in COVID-19 patients with decreased lymphocytes and increased neutrophils. Levels of lymphocytes and neutrophils and abundances of intestinal bacteria correlated with the severity of COVID-19.

## INTRODUCTION

The 2019 coronavirus disease (COVID-19) is a huge challenge for the global healthcare system. Severe acute respiratory syndrome-Coronavirus 2 (SARS-CoV-2) infection affects multiple organs, and patients with severe symptoms have poor survival outcomes (1 – 5). Early identification of patients with severe disease courses is of great significance.

Among mild COVID-19 patients, 23.3% of patients had only digestive symptoms with diarrhea, and 33.5% had both digestive and respiratory symptoms (6). Studies have shown that the duration of fever and dyspnea in COVID-19 patients with diarrhea is significantly longer than those without diarrhea (7). SARS-CoV-2 infects host cells through the functional receptor, angiotensin-converting enzyme 2 (ACE2) (8), which explains the gastrointestinal symptoms of COVID-19 patients, as ACE2 is highly expressed in the gut. ACE2, essential for the stable expression of intestinal neutral amino acid transporters, regulates innate immunity and modulates intestinal microbial homeostasis (9). Studies have shown that SARS-CoV-2 interacts with gut microbiome. Fecal samples with high SARS-CoV-2 infectivity had higher abundances of *Collinsella aerofaciens*, *Collinsella tanakaei*, *Streptococcus infantis*, and *Morganella morganii* (10). Bacterial dysbiosis persisted even after the removal of SARS-CoV-2 (identified from throat swabs), and the relieving of respiratory symptoms, particularly multiple species from the *Bacteroidetes* phylum, continued to decrease during the patients’ hospitalization (11). Patients with COVID-19 have an increased abundance of opportunistic pathogens such as *Prevotella*, *Enterococcus*, *Enterobacteriaceae*, or *Campylobacter*, and depletion of beneficial symbiotic bacteria such as *Faecalibacterium prausnitzii* and *Clostridium* species (12).

The gut microbiome has been found to play an important role in the severity of COVID-19. Fecal bacteria of severe and fatal COVID-19 patients clustered together and separated from mild courses and symptomatic pneumonia controls (13). Patients with severe disease progression had significantly increased abundances of *Clostridium innocuum*, *Ruthenibacterium lactatiformans*, and *Alistipes finegoldii* and decreased abundances of *Faecalibacterium prausnitzii*, *Blautia luti*, *Dorea longicatena*, *Gemmiger formicilis*, and *Alistipes putredinis* (13). The baseline abundance of *Coprobacillus*, *Clostridium ramosum*, and *Clostridium hathewayi* positively correlated with COVID-19 severity, while abundance of *Faecalibacterium prausnitzii* negatively correlated with disease severity (11). The relative abundance of *Proteobacteria* increased gradually from 3% in outpatients to 12% and 14% in wards and ICU COVID-19 patients separately, while the ratio of *Firmicutes/Bacteroidetes* and the abundance of butyrate-producing bacteria gradually declined (14).

Many studies have shown that a “cytokine storm” occurs in severe COVID-19 patients, mainly due to increased production of cytokines and chemokines, leading to high levels of viral-induced inflammatory injury (15). K18-hACE2 mice infected intranasally with SARS-CoV-2 developed gut microbiome dysbiosis and observed translocation of bacteria into the blood (16). Severe COVID-19 patients have been found with significantly increased levels of lipopolysaccharide-binding protein, accompanied by elevated inflammatory factors and immune cells (17). COVID-19 patients with low level of *Coprococcus comes* had upregulated genes of viral transcription and apoptotic signal in peripheral blood mononuclear cells, and COVID-19 patients with high levels of *Enterococcus faecium* had upregulated genes of neutrophil degranulation and defense response to Gram-negative bacterium in PBMCs (18). Neutrophil-to-lymphocyte ratio (NLR) is established as a marker of immune system homeostasis (19). High NLR (>6.1) at admission was a risk predictor of death in patients with COVID-19 (20). Multivariate analysis showed that for every unit increase in NLR, the risk of in-hospital mortality increased by 8% (21).

In this study, we described the characteristics of the gut bacteria of COVID-19 patients and compared the differences in intestinal microbiota between COVID-19 patients with and without lymphocyte decline and COVID-19 patients with and without neutrophil elevation.

## RESULTS

### Alterations of gut microbiota in COVID-19 patients

**Fig 1:**
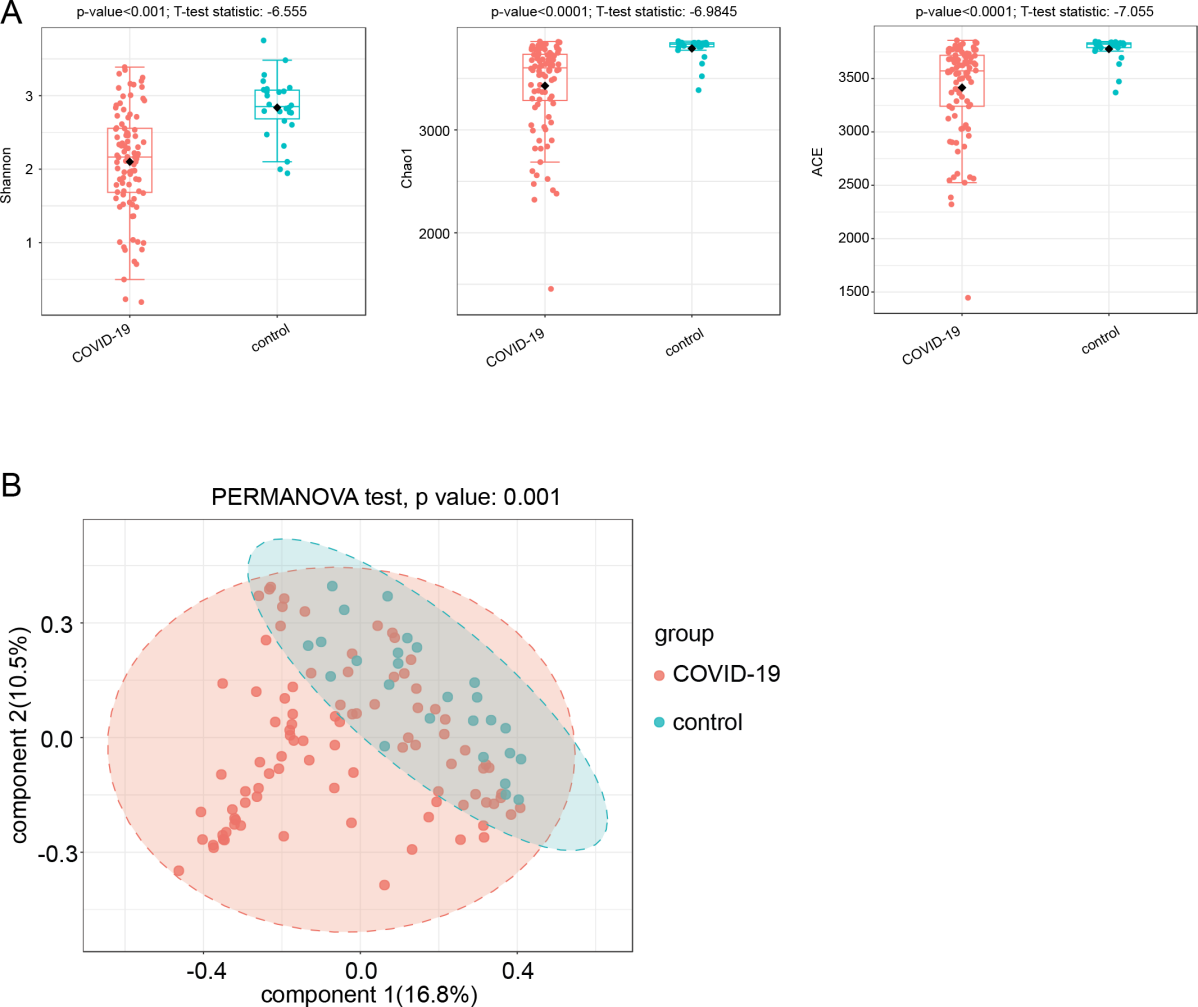
Intestinal bacterial diversities of COVID-19 patients and control individuals. (**A**) Microbial α-diversity measured by Shannon, Chao1, and ACE indexes. (**B**) Microbial β-diversity measured by PCoA.

A total of 92 adults who tested positive for SARS-CoV-2 and 25 healthy controls were included in our study. We obtained high-quality fecal DNA from these patients and healthy controls for metagenomic analysis. The microbial diversities were decreased in COVID-19 patients compared with controls, measured by Shannon, ACE, and Chao1 indexes and principal coordinate analysis (PcoA) analysis (Fig. 1).

### COVID-19 patients with decreased lymphocytes and/or increased neutrophils have unique fecal bacterial profiles

**Fig 2:**
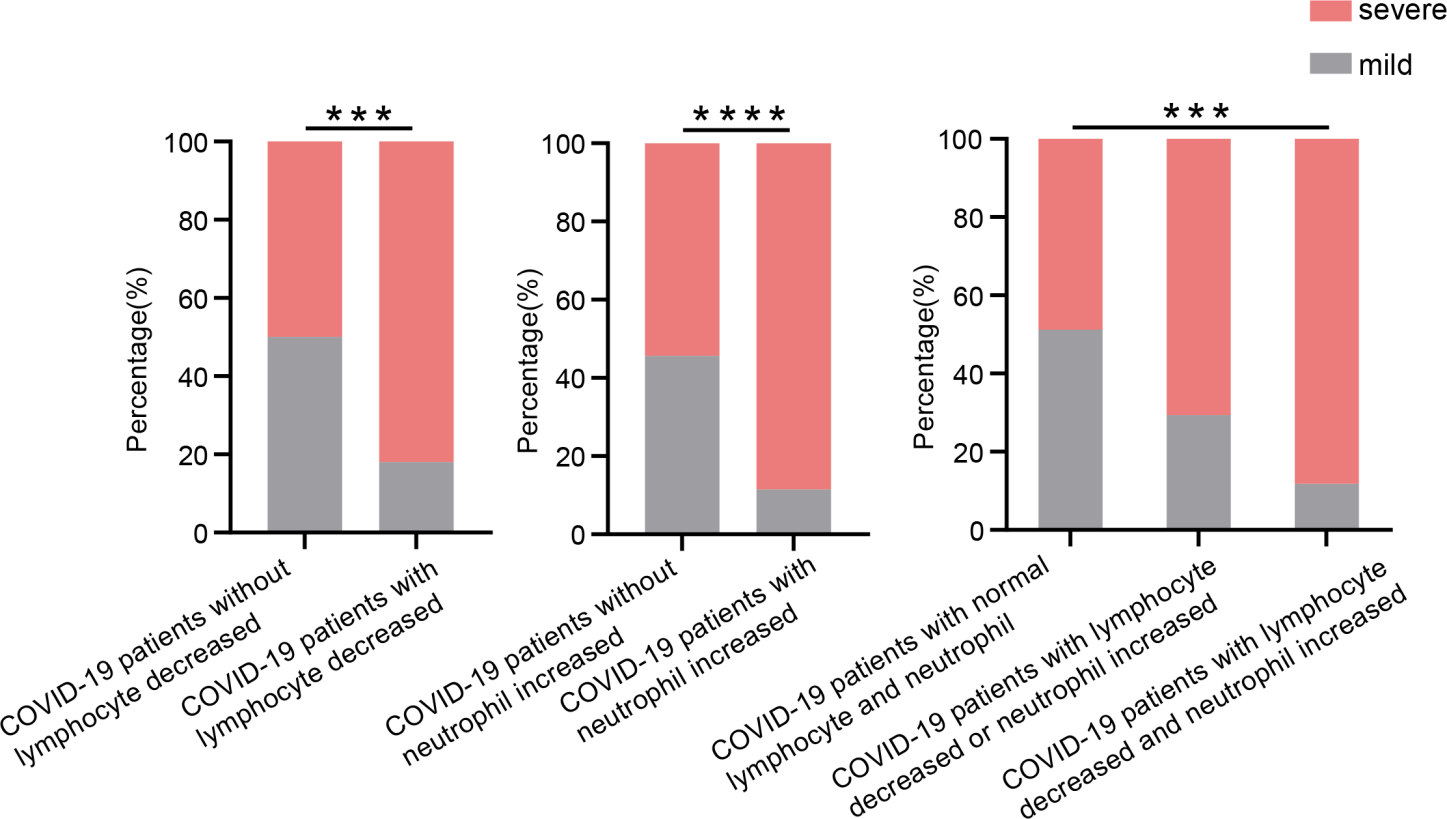
COVID-19 patients with decreased lymphocytes and increased neutrophils have higher rates of severe individuals. The proportion of severe patients in each group was analyzed and compared by Chi-square test. ***P < 0.001 and ****P < 0.0001.

**Fig 3:**
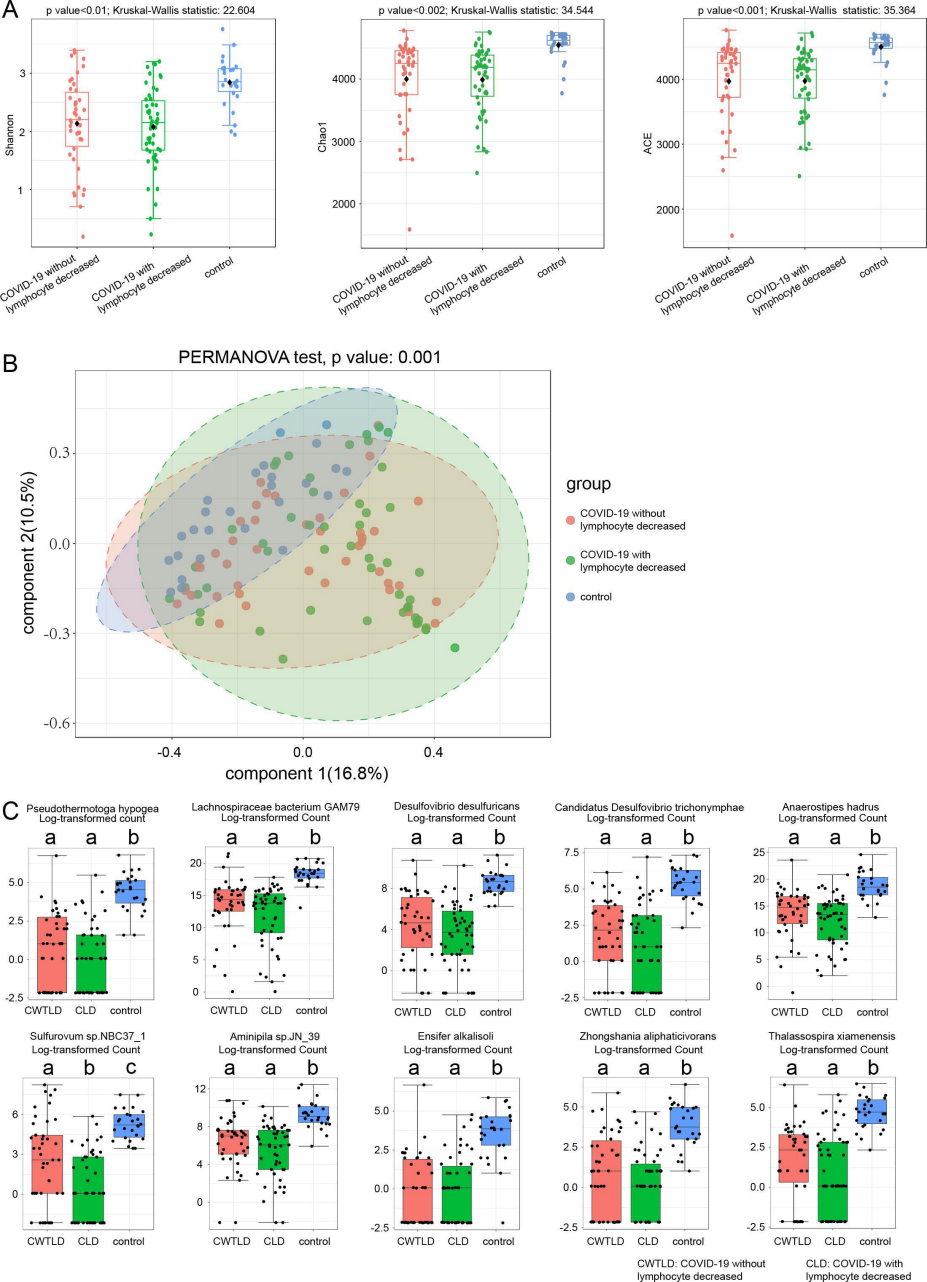
Characteristics of fecal bacteria among COVID-19 patients with and without decreased lymphocytes and healthy controls. (**A**) Microbial α-diversity measured by Shannon, Chao1, and ACE indexes. (**B**) Microbial β-diversity measured by PCoA. (**C**) The top 10 changed species measured by Kruskal-Wallis test. There were differences between a and b, a and c, and b and c measured by Dunnett’s *t* test.

**Fig 4:**
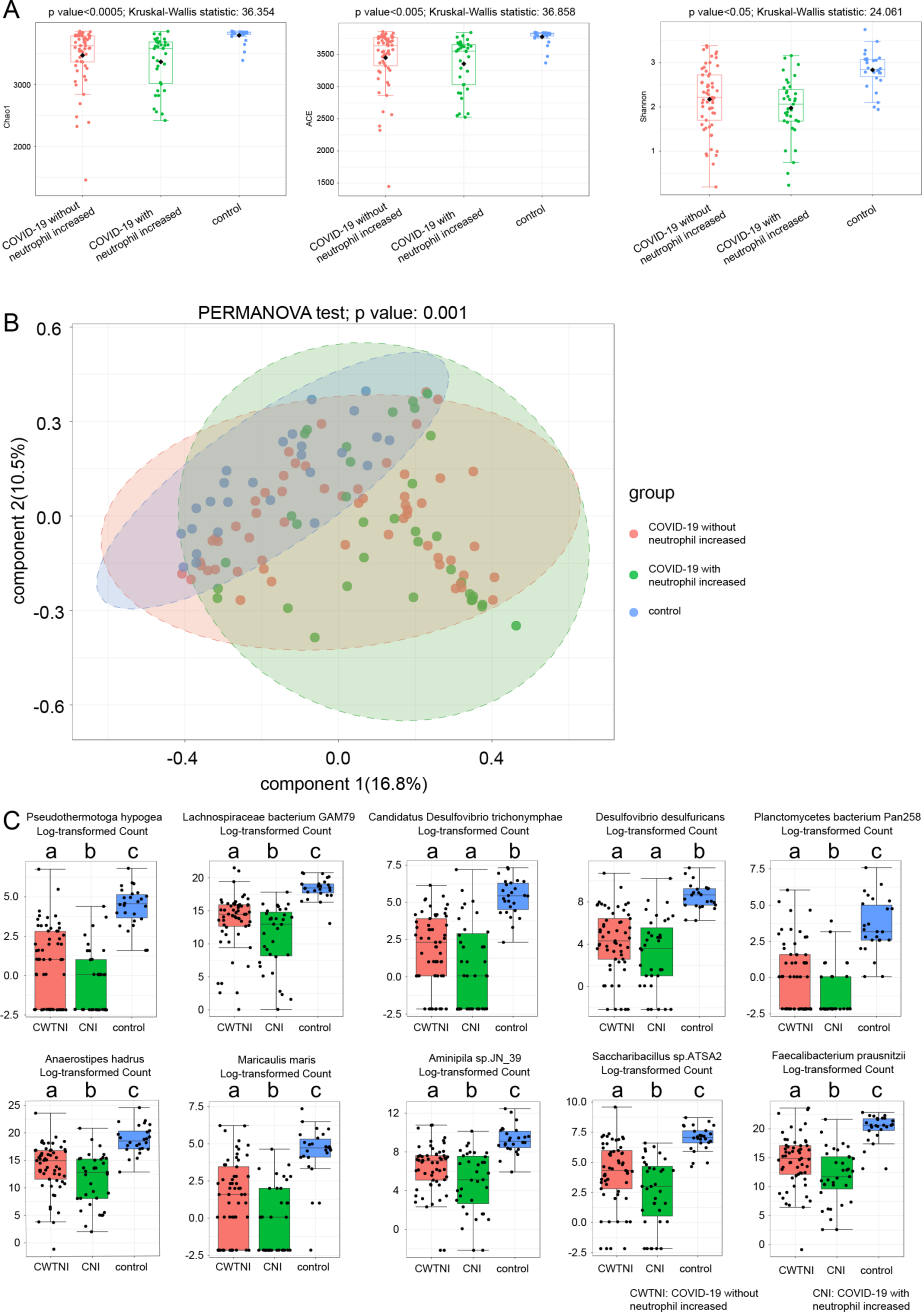
Characteristics of fecal bacteria among COVID-19 patients with and without increased neutrophils and healthy controls. (**A**) Microbial α-diversity measured by Shannon, Chao1, and ACE indexes. (**B**) Microbial β-diversity measured by PCoA. (**C**) The top 10 changed species measured by Kruskal-Wallis test. There were differences between a and b, a and c, and b and c measured by Dunnett’s *t* test.

The age, percentage of males, and percentages of all morbidities were not different between COVID-19 patients without and with decreased lymphocytes (Table 1). The age, percentage of males, and percentages of hypertension, diabetes, chronic renal disease, and cancer were also not different between COVID-19 patients without and with increased neutrophils, except that the percentage of patients with coronary heart disease was higher in those with increased neutrophils than those without (Table 1). The proportions of severe patients increased significantly in COVID-19 patients with decreased lymphocytes and/or increased neutrophils compared with COVID-19 patients with normal lymphocytes and neutrophils (Fig. 2). There were significant differences in bacterial α-diversities measured by Shannon, ACE, and Chao1 indexes among the control group and COVID-19 patients with and without lymphocytes declined. Obvious differences in bacterial β-diversities among the three groups were also observed (Fig. 3A and B). Similarly, Fig. 4A and B show the differences in bacterial diversity among the control group and COVID-19 patients with or without elevated neutrophils.

**TABLE 1:** Characteristics of COVID-19 patients without and with decreased lymphocytes or increased neutrophils

Characteristics	COVID-19 patients			COVID-19 patients		
	Without decreased lymphocytes	With decreased lymphocytes	*P* value	Without increased neutrophils	With increased neutrophils	*P* value
	*N* = 42	*N* = 50		*N* = 57	*N* = 35	
Age, years (median ± SD)	59.3 ± 11.11	62.1 ± 11.21	0.232	59.5 ± 10.53	62.9 ± 12.06	0.160
Male, *n* (%)	19 (45.2)	24 (48.0)	0.791	28 (49.1)	15 (42.9)	0.559
Comorbidities [*n*, (%)]						
Hypertension	16 (38.1)	17 (34.0)	0.683	21 (26.8)	12 (34.3)	0.804
Diabetes	5 (11.9)	11 (22.0)	0.203	8 (14.0)	8 (22.9)	0.278
Coronary heart disease	1 (2.4)	6 (12.0)	0.083	1 (1.8)	6 (17.1)	0.011
Chronic renal disease	0 (0.0)	0 (0.0)	–^*a*^	0 (0.0)	0 (0.0)	–
Cancer	0 (0.0)	1 (2.0)	1.000	0 (0.0)	1 (2.9)	0.380

^*a*^ The *P* value could not be calculated because there were no patients with chronic renal disease.

The abundances of *Pseudothermotoga hypogea*, *Lachnospiraceae bacterium GAM79*, *Desulfovibrio desulfuricans*, *Candidatus Desulfovibrio trichonymphae*, *Anaerostipes hadrus*, *Sulfurovum* sp. *NBC37_1*, *Aminipila* sp. *JN_39*, *Ensifer alkalisoli*, *Zhongshania aliphaticivorans*, and *Thalassospira xiamenensis* species were decreased in COVID-19 patients with or without decreased lymphocytes compared with control subjects (Fig. 3C). *Sulfurovum* sp. *NBC37_1* was enriched in COVID-19 patients without decreased lymphocytes than those with decreased lymphocytes. The abundances of *Pseudothermotoga hypogea*, *Lachnospiraceae bacterium GAM79*, *Candidatus Desulfovibrio trichonymphae*, *Desulfovibrio desulfuricans*, *Planctomycetes bacterium Pan258*, *Anaerostipes hadrus*, *Maricaulis maris*, *Aminipila* sp. *JN_39*, *Saccharibacillus* sp. *ATSA2*, and *Faecalibacterium prausnitzii* species were decreased in COVID-19 patients with or without elevated neutrophils compared with control subjects (Fig. 4C). Except for *Candidatus Desulfovibrio trichonymphae* and *Desulfovibrio desulfuricans*, the rest of the species significantly declined in COVID-19 patients with increased neutrophils than those without increased neutrophil.

**Fig 5:**
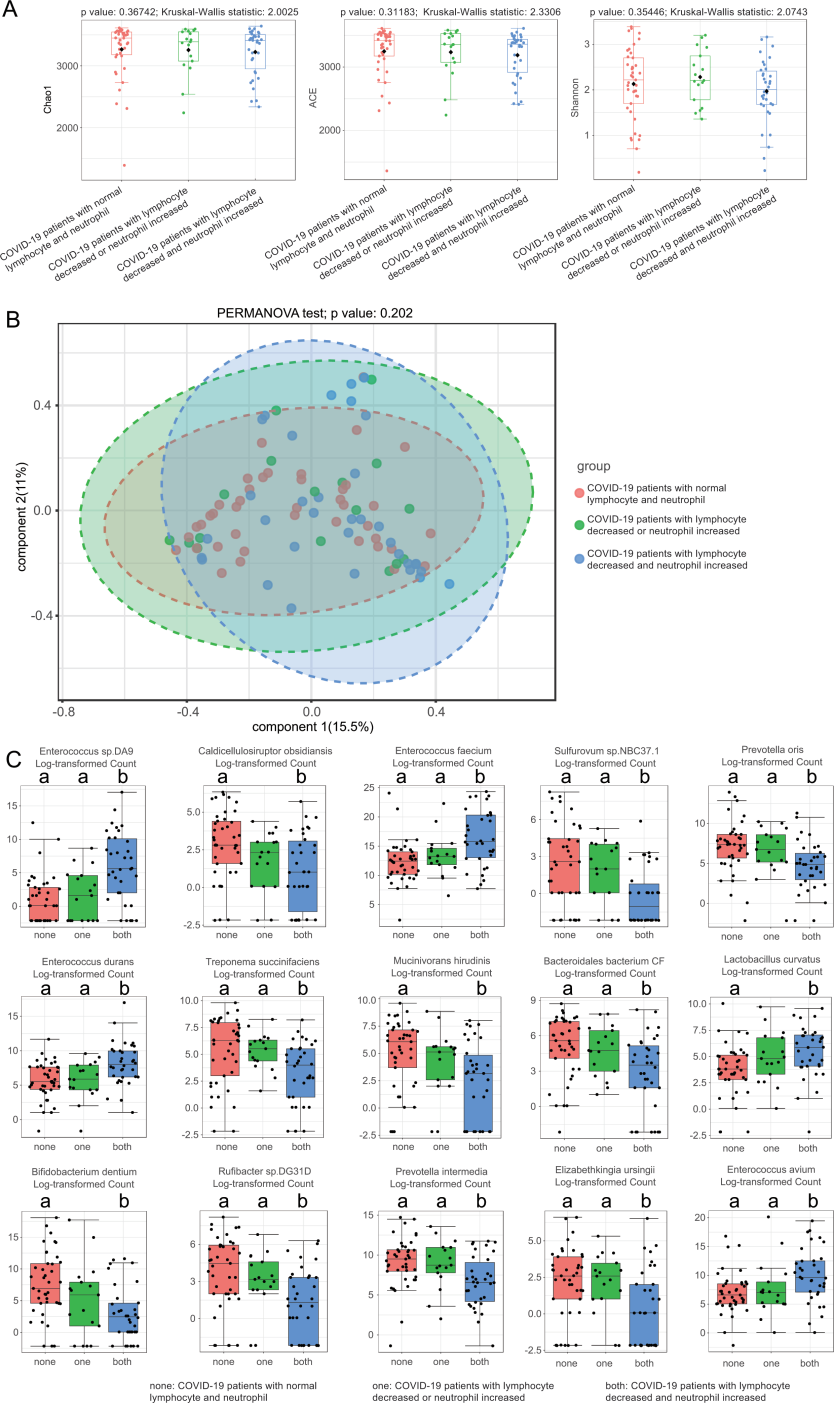
Characteristics of fecal bacteria among COVID-19 patients with and without decreased lymphocytes or increased neutrophils. (**A**) Microbial α-diversity measured by Shannon, Chao1, and ACE indexes. (**B**) Microbial β-diversity measured by PCoA. (**C**) The top 15 changed species measured by Kruskal-Wallis test. There was a difference between a and b measured by Dunnett’s *t* test.

There was no difference in bacterial diversity among COVID-19 patients with normal lymphocytes and neutrophils and COVID-19 patients with decreased lymphocytes and/or increased neutrophils (Fig. 5A and B). The abundances of *Enterococcus* sp. *DA9*, *Enterococcus faecium*, *Enterococcus durans*, and *Enterococcus avium* were the highest in COVID-19 patients with decreased lymphocytes and increased neutrophils (both group) than in COVID-19 patients with normal lymphocytes and neutrophils (none group) and in COVID-19 patients with decreased lymphocytes or increased neutrophils (one group) (Fig. 5C). The abundances of *Sulfurovum* sp. NBC37.1, *Prevotella oris*, *Treponema succinifaciens*, *Bacteroidales bacterium CF*, *Rufibacter* sp. *DG31D*, *Prevotella intermedia*, and *Elizabethkingia ursingii* were the lowest in the “both group” than the “none group” and “one group.” Especially, the abundance of *Bifidobacterium dentium* was obviously decreased in the “both group” compared to the “none group,” but there were no differences with the “one group.”

### Levels of lymphocytes and neutrophils combined with abundances of bacteria perfectly predicted disease severity in COVID-19 patients

**Fig 6:**
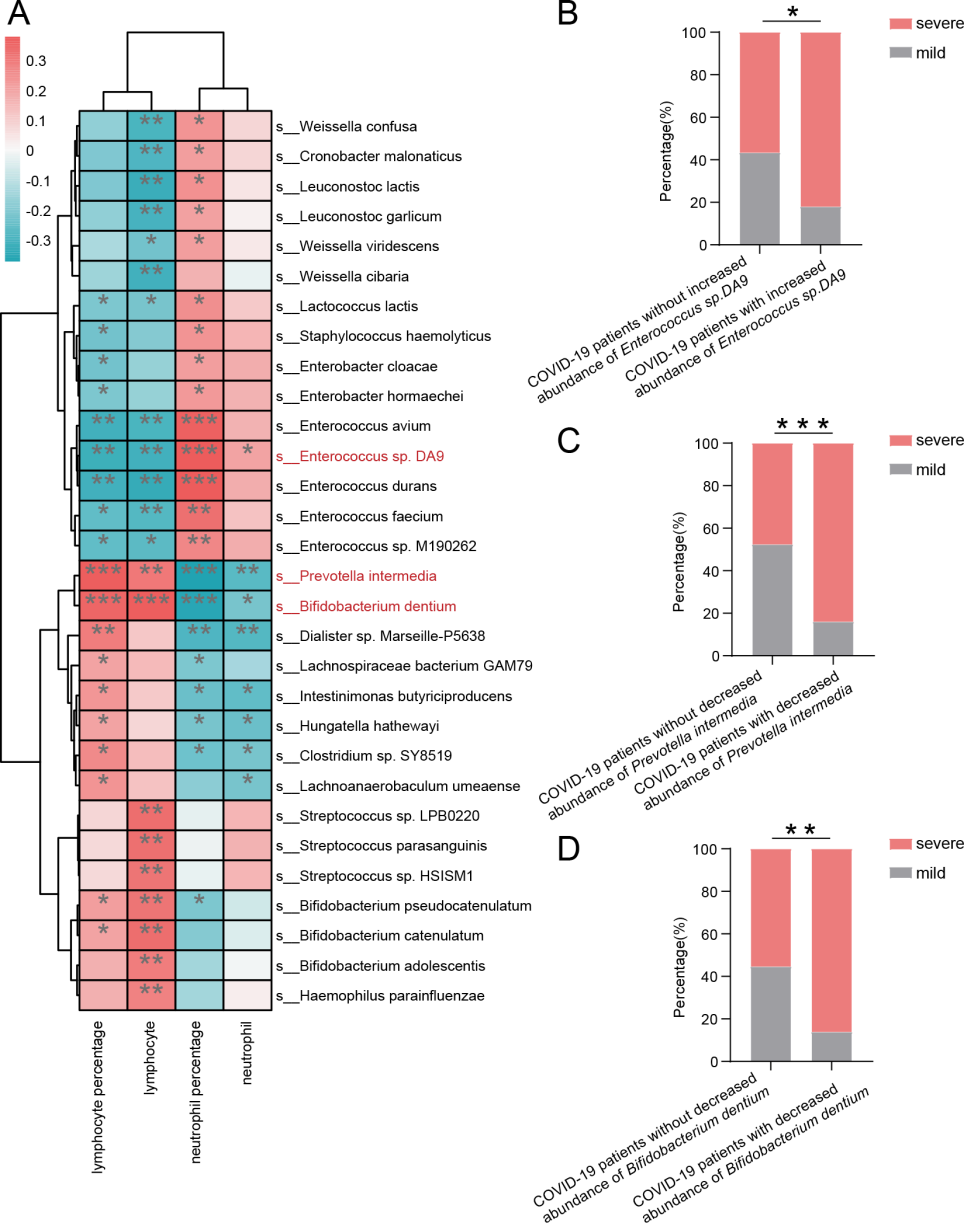
Fecal bacteria correlate with the disease severity of COVID-19 patients. (**A**) Correlation of bacteria with the levels of lymphocyte percentage, lymphocyte, neutrophil percentage, and neutrophil. (**B**) The proportions of severe individuals in COVID-19 patients with and without increased abundance of *Enterococcus* sp. *DA9*. (**C**) The proportions of severe individuals in COVID-19 patients with and without decreased abundance of *Prevotella intermedia*. (**D**) The proportions of severe individuals in COVID-19 patients with and without decreased abundance of *Bifidobacterium dentium*. *P < 0.05, **P < 0.005, and ***P < 0.0005.

**Fig 7:**
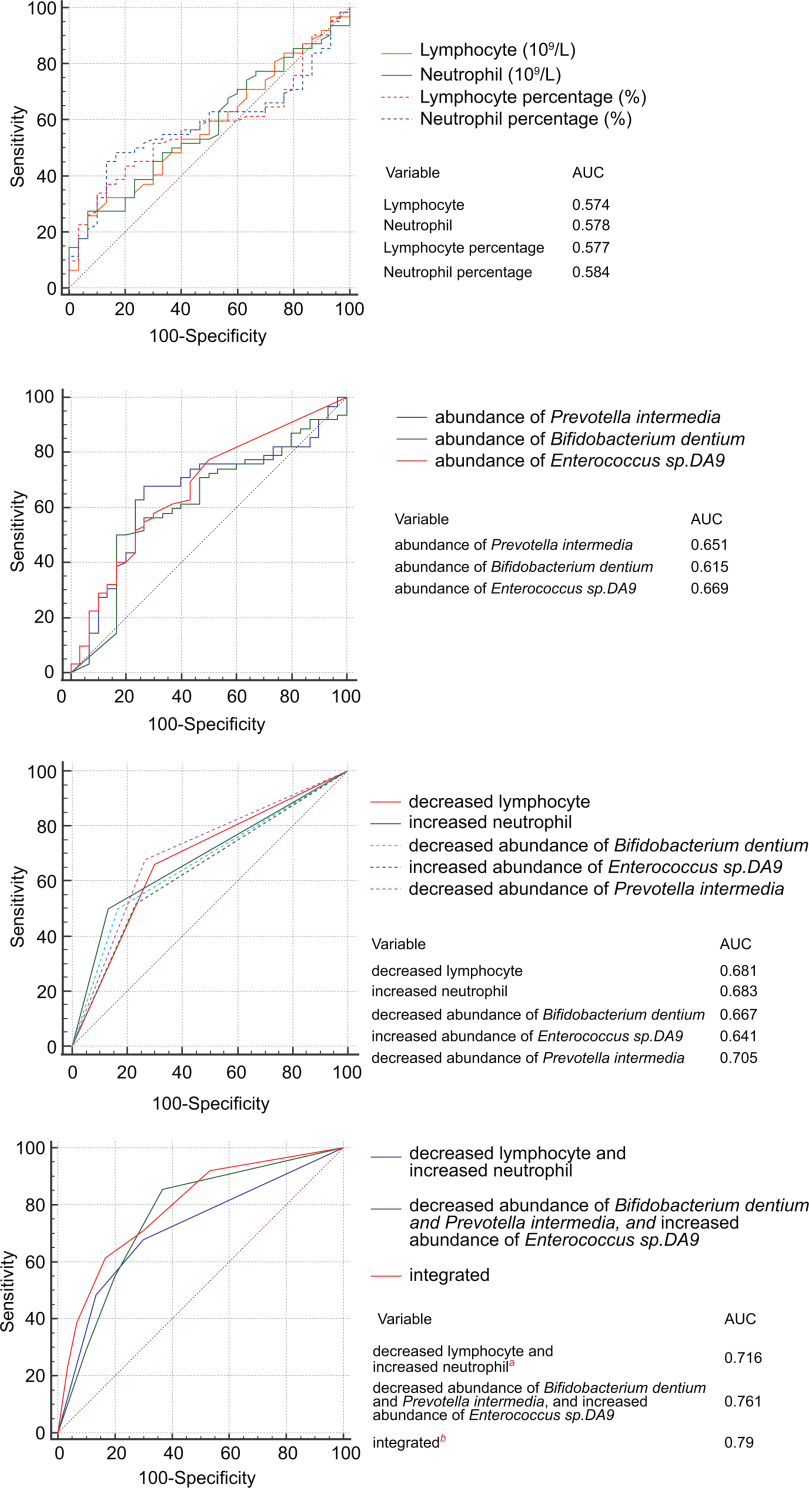
Prediction of disease severity by levels of lymphocytes and leukocytes combined with abundances of bacteria. (**A**) ROC curves of levels of lymphocyte percentage, lymphocytes, neutrophil percentage, and neutrophils to predict the disease severity of COVID-19 patients. (**B**) ROC curves of abundances of *Prevotella intermedia, Bifidobacterium dentium*, and *Enterococcus* sp. *DA9* to predict the disease severity of COVID-19 patients. (**C**) ROC curves of decreased lymphocytes, increased neutrophils, decreased abundance of *Bifidobacterium dentium* and *Prevotella intermedia,* and increased abundance of *Enterococcus* sp. *DA9* to predict the disease severity of COVID-19 patients. (**D**) ROC curves of decreased lymphocytes and increased neutrophils, changed abundances of bacterial species, and the integrated indexes to predict the disease severity of COVID-19 patients. There was a difference between a and b.

Correlation analysis of counts and percentages of lymphocytes and neutrophils and fecal abundances of bacteria showed that abundance of *Enterococcus* sp. *DA9* negatively related to the count and percentage of lymphocytes and positively related to the count and percentage of neutrophils, while abundances of *Prevotella intermedia* and *Bifidobacterium dentium* were positively related to the count and percentage of lymphocytes and negatively related to the count and percentage of neutrophils (Fig. 6A). COVID-19 patients with increased abundance of *Enterococcus* sp. *DA9* and decreased abundance of *Prevotella intermedia* or *Bifidobacterium dentium* were more likely to develop severe disease (Fig. 6B through D). Abundances of *Prevotella intermedia*, *Bifidobacterium dentium*, and *Enterococcus* sp. *DA9* had an advantage in predicting the disease severity of COVID-19 patients over counts and percentages of lymphocytes and neutrophils, as the areas under the receiver operating characteristic (ROC) curve of abundances of *Prevotella intermedia*, *Bifidobacterium dentium*, and *Enterococcus* sp. *DA9* were higher (Fig. 7). The predictive powers were also considerable when the level of lymphocytes and abundances of *Bifidobacterium dentium* or Prevotella *intermedia*declined and the level of neutrophils and abundance of *Enterococcus* sp. *DA9* increased to predict the disease severity of COVID-19 patients. The combination of changed lymphocytes and neutrophils and bacterial abundances had the greatest advantage in predicting the severity of COVID-19 patients.

## DISCUSSION

Consistent with previous studies, there was a significant decrease in the α-diversity of intestinal bacteria in COVID-19 patients compared to non-COVID-19 controls (22). The changes in intestinal flora composition were significantly associated with disease severity and prognosis of COVID-19 patients. Cytokines, metabolites, and pathobionts, such as *Enterococcus*, have been modeled to predict the severity of COVID-19 patients (23). *Enterococcus*, one of the common opportunistic pathogens, was found to increase in critically ill COVID-19 patients (24). The area under the curve (AUC) of predicting severe patients by the increased abundance of *Enterococcus* sp. *DA9* was 0.641.

The 4C score performed well in identifying the risk of death in COVID-19 patients, and the ROC was 0.79 (25). Delayed or overall inhibition of the type I IFN response to SARS-CoV-2 virus invasion may develop into a life-threatening high-inflammatory disease as the immune system struggles to limit viral replication and get rid of dead cells, which triggers a more vigorous immune response (26). *Prevotella* has been reported to induce the production and accumulation of Th17 cells in the colon after colonizing the intestine, and an increased abundance of *Prevotella* is associated with Th17-mediated mucosal inflammation (27). However, we found that the abundance of *Prevoteria intermedia* was decreased in COVID-19 patients and inversely associated with disease severity. Genera from the *Bifidobacteriaceae* and *Lachnospiraceae* families that have potential immunomodulatory capacity were found to be decreased in COVID-19 patients (28). In our study, *Lachnospiraceae_bacterium_GAM79* was found to be significantly decreased in COVID-19 patients compared with healthy controls. While *Bifidobacterium pseudocatenulatum* and *Bifidobacterium catenulatum* were obviously reduced in severe COVID-19 patients in contrast to mild COVID-19 patients, *Bifidobacterium dentium* showed a positive correlation with levels of lymphocytes, a negative correlation with levels of leukocytes, and related with disease severity in COVID-19 patients.

Short-chain fatty acid (SCFA) and L-isoleucine biosynthesis of the intestinal microbiota are impaired in severe/critical COVID-19 patients compared to non-COVID-19 controls, and the injuries persist even after disease remission (29). Abundances of short-chain fatty acid-producing species, such as *Butyrivibrio hungatei*, *Butyrivibrio proteoclasticus*, *Butyrivibrio fibrisolvens*, *Roseburia hominis*, *Blautia* sp. *YL58*, *Faecalibacterium prausnitzii*, and *Eubacterium cellulosolvens*, were dramatically decreased in COVID-19 patients. Similar results were reported by Nagata et al. (30) and He et al. (31). Members of *Lachnospiraceae*, known as producers of SCFAs (32), were poor in COVID-19 patients with decreased lymphocytes and increased neutrophils (Fig. 3 and 4). SCFAs are energy substrates of intestinal epithelial cells and maintain intestinal immune homeostasis (33). A decrease in the abundance of *Lachnospiraceae* in severe COVID-19 patients is then expected. Decreased *Lachnospiraceae* can facilitate the translocation of lipopolysaccharide (LPS) through the hyperpermeable gut, resulting in an “inflammatory cytokine storm” of the circulatory system. SCFAs have been proven to promote IL-10 production of mucosal Treg cells and inhibit the production of inflammatory cytokines, TNF-α, MCP-1, IL-6, IL-8, and IL-12, by macrophages, thus limiting intestinal inflammatory response (34). Dietary fiber supplements, which are fermented by intestinal flora to produce SCFAs, might enhance the host immunity and reduce the intestinal inflammatory response of COVID-19 patients. Fermented vegetables rich in sulforaphane and *Lactobacilli* have powerful antioxidant effects and contribute to lower COVID-19 mortality (35).

Zhang et al. have reported that counts and percentages of neutrophils, lymphocytes, and monocytes were significantly different among COVID-19 patients with various disease severity (36). As the disease worsened, neutrophils gradually increased and lymphocytes gradually decreased, and the AUC of lymphocytes to predict the disease severity of COVID-19 patients was 0.718. Elevated neutrophil-to-lymphocyte ratio was significantly associated with illness severity, and NLR exhibited the largest area under the curve at 0.841, with the highest specificity (63.6%) and sensitivity (88%) (37). Xu et al. found that as the disease progressed, the T lymphocyte count dropped, and the T cells and their CD3^+^, CD4^+^, and CD8^+^ subsets and B cells were significantly lower in non-survival patients than those of other patients (38). Using lymphocyte and granulocyte-related parameters to build the model can well predict the disease severity and survival of COVID-19 patients. With the optimal cut-off score of 30.6 obtained by ROC curve, the sensitivity and specificity of detection were 82.0% and 82.5%, respectively (39).

Increased absolute and relative neutrophil abundance and reduced overall T and B lymphocytes have been found in more severe/critical COVID-19 patients (40). In the early stages of infection, lymphocytes and lymphocyte subsets (T cells, CD4^+^, and CD8^+^ T cell subsets) were significantly lower in patients with severe COVID-19 and severe influenza A than in healthy controls (41). Prozan et al. compared the prognostic value of NLR among COVID-19, influenza and respiratory syncytial virus infection and showed that NLR was associated with poor clinical outcome only in the COVID-19 group (42). For COVID-19 patients, after adjusting for age, sex, and Charlson comorbidity score, multivariate logistic regression found that high NLR (>6.82) was still a poor prognostic factor. NLR < 3.2 could independently distinguish COVID-19 from other upper respiratory tract infections including influenza (OR: 4.23, *P* = 0.0494, AUC: 0.673), and its efficacy was greatly improved (AUC: 0.840) when combined with the monocyte distribution width (≥20) (43).

In conclusion, the gut microbiome and white blood cell seem to contribute to the course and severity of COVID-19. Disordered gut microbiome, decreased lymphocytes, and increased neutrophils may be predictive biomarkers of COVID-19 severity.

## MATERIALS AND METHODS

### Patients enrolled

Ninety-two hospitalized COVID-19 patients with laboratory-confirmed SARS-CoV-2 infection and 22 control individuals were included in the study. SARS-CoV-2 infection was diagnosed by reverse-transcriptase polymerase chain reaction assay using respiratory tract samples. Enrolled COVID-19 patients were admitted to Union Hospital (Wuhan, China) from January to March 2020. The control group was subjects recruited from the hospital isolation site during the same period with respiratory symptoms and confirmed with no SARS-CoV-2 infection.

### Classification of COVID-19 patients

Disease severity was decided according to the seventh edition of the Chinese diagnostic criteria of COVID-19 Diagnosis and Treatment Protocol (44). Patients who met any of the following criteria were defined as severe types: respiratory rate 30 breaths/minute; oxygen saturation ≤ 93% at a rest state; and arterial partial pressure of oxygen (PaO_2_)/ oxygen concentration (FiO_2_) ≤ 300 mmHg. COVID-19 patients with decreased lymphocytes were defined as the absolute count (<1.1 × 10^9^/L) or percentage (<20%) of lymphocytes was less than the lower limit of normal. COVID-19 patients with increased neutrophils were defined as the absolute count (>6.3 × 10^9^/L) or percentage (>75%) of neutrophils was more than the higher limit of normal.

### Fecal metagenome sequencing

After collection, fecal samples were immediately sent to the laboratory for inactivation at 95°C for 30 minutes and then sent to the Wuhan BGI Laboratory for metagenomics testing. TIANamp fecal DNA Kit (DP328, Tiangen Biotechnology) was used to extract total fecal DNA according to the manufacturer’s instructions. DNA libraries were constructed through end repair, adding A to tails, purification, and PCR amplification. After being qualified by the Agilent 2100 bioanalyzer (Agilent Technologies, Santa Clara, CA, USA), DNA libraries were sequenced on the BGISEQ platform (MGI BGISEQ-50, BGI Wuhan Clinical Laboratories, Wuhan, China).

Raw sequence readings were filtered and qualified by Trimmomatic v0.39 according to the following criteria: (i) pruning bases with a quality score below 20 and (ii) removing reads shorter than 35 base pairs. Contaminating human reads were filtered using Kneaddata (reference database: GRCh38/hg38) with default parameters. Then, microbial taxonomies were profiled by MetaPhlAn3.

### Bioinformatic analysis

α-diversity of the intestinal microbiota was measured by the Shannon, Chao1, and ACE indexes, and the β-diversity of the intestinal microbiota was analyzed by PCoA (using the MicrobiomeAnalyst 2.0 tool). Species with significant differences in multi-groups were tested by Kruskal-Wallis, and Dunnett’s *t* test was further used to analyze the difference between the two groups.

### Receiver operating characteristic analysis

The AUC were calculated to evaluate the discriminating power of each indicator in identifying severe COVID-19 patients. ROC analysis was performed with MedCalc software, and differences in AUC were compared by the DeLong et al. (45) methodology. The cut-off of decreased abundance of *Prevotella intermedia*, *Bifidobacterium dentium* and increased abundance of *Enterococcus* sp. *DA9* was measured by the Youden index.

### Statistical analysis

Continuous variables were represented by mean ± standard deviation, and categorical variables were described by number and percentage. Two-tailed Student’s *t* tests were used for continuous variables, and the Chi-square test or Fisher’s Exact test was used for categorical variables to detect the differences. A *P*-value of less than 0.05 was defined as a significant difference.

## Data Availability

Data supporting the results of this study have been presented in the paper, and more data can be requested from the corresponding authors.
